# Reporting of statistically significant results at ClinicalTrials.gov for completed superiority randomized controlled trials

**DOI:** 10.1186/s12916-016-0740-1

**Published:** 2016-11-30

**Authors:** Agnes Dechartres, Elizabeth G. Bond, Jordan Scheer, Carolina Riveros, Ignacio Atal, Philippe Ravaud

**Affiliations:** 1 Centre de Recherche Epidémiologie et Statistique, INSERM U1153, Paris, France; 2 Centre d’Epidémiologie Clinique, Hôpital Hôtel-Dieu, Assistance Publique-Hôpitaux de Paris, Paris, France; 3 Faculté de Médecine, Université Paris Descartes, Sorbonne Paris Cité, Paris, France; 4 Cochrane France, Paris, France; 5 Department of Epidemiology, Mailman School of Public Health, Columbia University, New York, NY USA

**Keywords:** Clinical trials, Reporting, Publication bias, Registration, Results, Transparency

## Abstract

**Background:**

Publication bias and other reporting bias have been well documented for journal articles, but no study has evaluated the nature of results posted at ClinicalTrials.gov. We aimed to assess how many randomized controlled trials (RCTs) with results posted at ClinicalTrials.gov report statistically significant results and whether the proportion of trials with significant results differs when no treatment effect estimate or *p*-value is posted.

**Methods:**

We searched ClinicalTrials.gov in June 2015 for all studies with results posted. We included completed RCTs with a superiority hypothesis and considered results for the first primary outcome with results posted. For each trial, we assessed whether a treatment effect estimate and/or *p*-value was reported at ClinicalTrials.gov and if yes, whether results were statistically significant. If no treatment effect estimate or *p*-value was reported, we calculated the treatment effect and corresponding *p*-value using results per arm posted at ClinicalTrials.gov when sufficient data were reported.

**Results:**

From the 17,536 studies with results posted at ClinicalTrials.gov, we identified 2823 completed phase 3 or 4 randomized trials with a superiority hypothesis. Of these, 1400 (50%) reported a treatment effect estimate and/or *p*-value. Results were statistically significant for 844 trials (60%), with a median *p*-value of 0.01 (Q1-Q3: 0.001–0.26). For the 1423 trials with no treatment effect estimate or *p*-value posted, we could calculate the treatment effect and corresponding *p*-value using results reported per arm for 929 (65%). For 494 trials (35%), *p*-values could not be calculated mainly because of insufficient reporting, censored data, or repeated measurements over time. For the 929 trials we could calculate *p*-values, we found statistically significant results for 342 (37%), with a median *p*-value of 0.19 (Q1-Q3: 0.005–0.59).

**Conclusions:**

Half of the trials with results posted at ClinicalTrials.gov reported a treatment effect estimate and/or *p*-value, with significant results for 60% of these. *p*-values could be calculated from results reported per arm at ClinicalTrials.gov for only 65% of the other trials. The proportion of significant results was much lower for these trials, which suggests a selective posting of treatment effect estimates and/or *p*-values when results are statistically significant.

## Background

Most patients assume that they are receiving evidence-based care made by well-informed medical practitioners. However, reporting bias within peer-reviewed literature makes that assumption difficult [1, 2]. Evidence of such reporting bias has been well documented [3–6], with many studies showing that trials with statistically significant results are more likely to be published and to be published more quickly than those with no statistical difference [3, 6–8]. Such reporting bias may affect the results of systematic reviews and meta-analyses toward more positive results [9, 10] and lead to erroneous decision-making with serious consequences for patients [11, 12].

To limit reporting bias, the International Committee of Medical Journal Editors (ICMJE) released a statement in 2005 that made trial registration a condition for publication [13–15]. A further step was achieved in 2007 with the US Food and Drug Administration Amendments Act (FDAAA 801) requiring phase 2–4 trials of FDA-approved drugs, devices, or biologics to post results on the federally funded registry ClinicalTrials.gov within 1 year of completion [16, 17]. According to the law, “a table of values for each of the primary and secondary outcome measures for each arm of the clinical trial, including the results of scientifically appropriate tests of the statistical significance of such outcome measures” should be posted [18]. However, it seems that many trials with results posted fail to report a an estimate of treatment effect or *p*-value.

In this study, we aimed to assess how many superiority clinical trials with results posted at ClinicalTrials.gov report statistically significant results and whether the proportion of significant results differed for trials with no treatment effect estimate or *p*-value posted.

## Methods

### Data sources

On 2 June 2015, one of the authors (EGB) searched ClinicalTrials.gov for “Studies with Results” in the Study Results field, then downloaded all records corresponding to these studies as excel and xml files. There was no limit on date. xml files were handled with R version R 3.2.3 (R Foundation for Statistical Computing, Vienna, Austria; https://www.R-project.org/) with the xml package.

### Identification of completed phase 3 or 4 clinical trials with a superiority hypothesis

Trials reported as “phase 3” or “phase 4” in the phase field, as “randomized” in the study design field, and as “completed” in the recruitment field were considered for inclusion. Trials not reporting these elements were excluded. We identified the number of arms reported in the study results and excluded single-arm trials as well as trials involving three or more arms so as to focus on comparisons between an experimental intervention and a control. We excluded non-inferiority and equivalence trials, which were identified if the keywords “non-inferiority,” “non-inferior,” “equivalence,” “bioequivalence,” “bio-equivalence,” or “equivalent” were present in the following fields: study design, endpoint classification, or study results. We manually verified that each trial excluded on the basis of these criteria met the definition of a non-inferiority trial (i.e., aiming to show that an experimental intervention is non-inferior to a control one) or equivalence trial (i.e., aiming to show that two interventions have therapeutic equivalence [19, 20]). We also excluded pharmacokinetic trials reported as a “bio-equivalence study” or “pharmacokinetics/dynamics study” in the endpoint classification field or based on information reported in the study results. We considered all other trials as superiority trials (i.e., aiming to show a statistical difference between two interventions [20]) and included them. The selection process was done by one reviewer (EGB) and checked by a second reviewer (JS). Any discrepancies were resolved by a third reviewer (AD).

### Extraction of data from ClinicalTrials.gov

The following characteristics were extracted from the records downloaded from ClinicalTrials.gov:

1. General characteristics: we collected the phase of the trial (phase 3 or 4), type of intervention assessed (e.g., drug, biological, or device), type of control (i.e., placebo, no treatment, or active control), sponsor, and collaborators. We considered that there was an industry sponsorship if the sponsor or one of the collaborators was industry. We also extracted sample size and primary completion date (i.e., date of final collection for the primary outcome).
2. Location: we collected the countries where the trial was conducted and whether the trial was conducted in a single country and the number of centers involved.
3. Results posted: we collected the date when results were first received and whether the study was likely subject to the FDAAA. This characteristic is based on an algorithm developed by the US National Library of Medicine. Then, for the first primary outcome reported in the Results section, we collected whether a treatment effect estimate and/or *p*-value was reported and, if yes, we extracted them.

### Reporting of statistically significant results for the first primary outcome reported

For each trial with a treatment effect estimate and/or *p*-value reported, we evaluated whether results reported were statistically significant or not. To do so, we relied on the *p*-value reported at ClinicalTrials.gov and considered *p*-values <0.05 as statistically significant. Five percent has been the most commonly used threshold for statistical significance in clinical intervention research [21]. When only a measure of treatment effect with 95% confidence interval (CI) was reported, we derived the *p*-value from the 95% CI using the formula reported by Altman and Bland [22].

### Calculation of *p*-values for trials with no treatment effect estimate or *p*-value reported

For trials with no treatment effect estimate or *p*-value reported, we calculated whenever possible the treatment effect estimate and corresponding *p*-value using the results per arm posted at ClinicalTrials.gov. This calculation was possible only for binary and continuous outcomes when sufficient data were reported at ClinicalTrials.gov. For binary outcomes, we calculated relative risk with 95% CI and the corresponding *p*-value from the number of events and number of participants analyzed reported per arm. For continuous outcomes, we calculated mean difference with 95% CI and the corresponding *p*-value from the mean and standard deviation (SD) or standard error (SE), which we transformed to SD as well as the number of participants analyzed reported per arm.

Extraction of results per arm from ClinicalTrials.gov and calculation of treatment effect estimate with 95% CI and the corresponding *p*-value was systematically performed by two reviewers working independently (JS and CR) using Revman 5.3 (Copenhagen: The Nordic Cochrane Centre, The Cochrane Collaboration, 2014). Then, all results were compared and any disagreement was resolved by consensus with the help of a senior researcher (AD) if needed. We also considered a *p*-value <0.05 as statistically significant.

Calculation of treatment effect estimate and corresponding *p*-value was not possible for censored outcomes (e.g., progression-free survival), repeated measurements of the outcome over time (e.g., change in mean bone mineral density from baseline assessed at 12, 24, and 36 months), other situations for which there were several observations or events per patient because these situations required individual patient data, or when there was no event in both groups.

### Statistical analysis

Categorical variables were described with frequencies (percentages) and quantitative variables with median (Q1-Q3). To assess the statistical significance of the results, we focused on *p*-values rather than on treatment effect estimates with 95% CI, and graphically represented the density plots of *p*-values for trials by whether a treatment effect estimate and/or *p*-value was reported or not. For this, we considered *p*-values <0.0001 equal to 0.0001.

We aimed to identify trial characteristics associated with reporting a *p*-value <0.05 for the first primary outcome at ClinicalTrials.gov. To do, so, we first compared trial characteristics by whether results were reported to be statistically significant or not using chi-square tests for categorical variables and Wilcoxon tests for continuous variables. Then, we used a multivariate logistic regression model to identify factors independently associated with the reporting of statistically significant results. All variables that were statistically significant on univariate analyses were entered into multivariate analysis except single-center, which was closely related to single-country status.

Statistical analysis involved use of SAS 9.3 (SAS Inst., Cary, NC, USA). The density plot was created with R 3.2.3 (R Foundation for Statistical Computing) using a syntax provided in a previous article [23].

## Results

### Identification of completed randomized trials with a superiority hypothesis

**Fig. 1:**
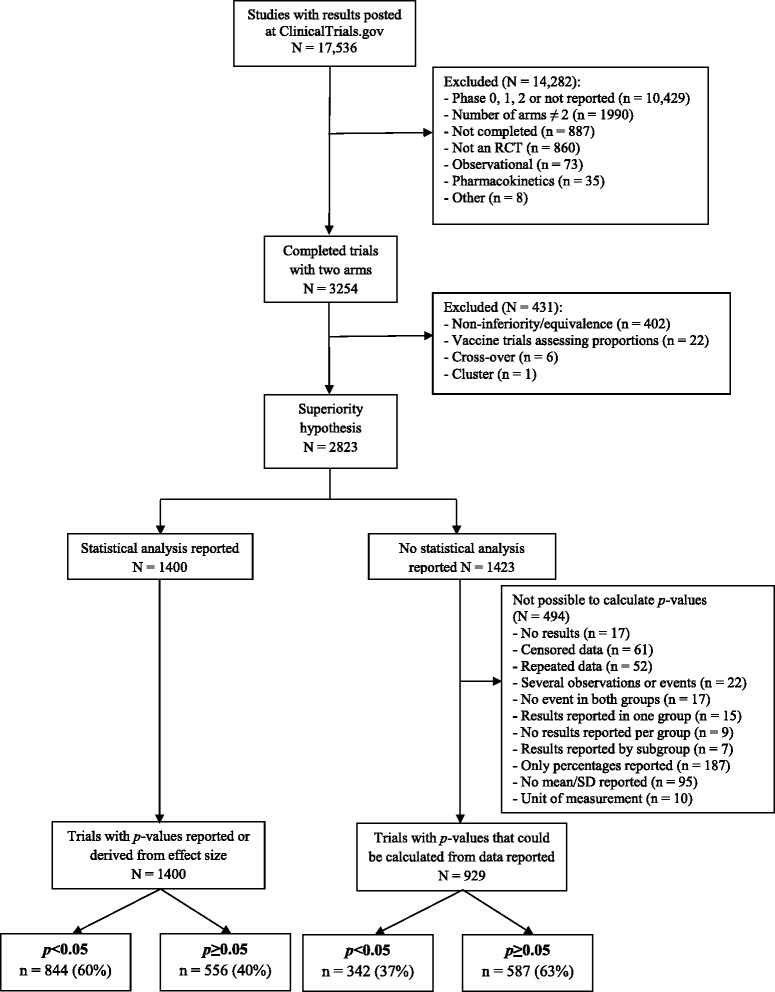
Flow chart of the selection of trials. *RCT* randomized controlled trial, *SD* standard deviation

Figure 1 shows the selection process. From the 192,175 studies registered at ClinicalTrials.gov on 2 June 2015, 17,536 had results posted, with 9640 (55%) likely to be subject to the FDAAA. We identified 2823 completed phase 3 or 4 randomized trials with a superiority hypothesis.

### Reporting of treatment effect estimate and/or *p*-value at ClinicalTrials.gov

Among the 2823 eligible trials, 1400 (50%) had a treatment effect estimate and/or *p*-value reported at ClinicalTrials.gov, and 1423 trials (50%) had only results per arm reported. Characteristics of trials by whether a treatment effect estimate and/or *p*-value was reported or not are reported in Table 1. Briefly, as compared with trials with no treatment effect estimate or *p*-value reported, those with a treatment effect estimate and/or *p*-value reported were more likely to be phase 3 trials (68% versus 51%), to have an inactive comparator (59% versus 36%), to have an industry sponsorship (83% versus 74%), to involve several countries (41% versus 21%) and several centers (66% versus 49%), to be subject to the FDAAA (68% versus 59%), and to have a larger median sample size (270 versus 156).

**Table 1:** Characteristics of completed phase 3 or 4 randomized clinical trials with results posted and a superiority hypothesis by whether a treatment effect estimate and/or *p*-value is reported at ClinicalTrials.gov

Characteristics	Categories	Trials with treatment effect estimate and/or *p*-value reported (N = 1400)	Trials with no treatment effect estimate or *p*-value reported (N = 1423)
Phase of the study	Phase 3	948 (68)	728 (51)
	Phase 4	452 (32)	695 (49)
Intervention type	Drug	1088 (78)	1014 (71)
	Mixed interventions	180 (13)	148 (10)
	Biological	48 (3)	96 (7)
	Device	40 (3)	97 (7)
	Other	44 (3)	68 (5)
Control group	Placebo or no treatment	822 (59)	516 (36)
	Active treatment	578 (41)	907 (64)
Sponsorship	Industry totally or partly	1162 (83)	1055 (74)
	Academic only	238 (17)	368 (26)
Countries	Single country	646 (46)	982 (69)
	Multiple countries	568 (41)	292 (21)
	Not reported	186 (13)	149 (10)
Location	At least one site in the USA	846 (61)	822 (58)
	No site in the USA	368 (26)	452 (32)
	Not reported	186 (13)	149 (10)
Subject to the FDAAA	Yes	951 (68)	843 (59)
	No	377 (27)	556 (39)
	Not reported	72 (5)	24 (2)
Centers	Multicenter	929 (66)	696 (49)
	Single center	285 (20)	578 (41)
	Not reported	186 (13)	149 (10)
Sample size	Median (Q1-Q3)	270 (116–520)	156 (60–378)
Time to results first received at ClinicalTrials.gov (month)	Median (Q1-Q3)	13.5 (11.7–26.4)	17.1 (12.0–32.3)

Data are presented as n (%) unless indicated. *FDAAA* US Food and Drug Administration Amendments Act, *IQR* Q1-Q3

### Reporting of statistically significant results for the first primary outcome

**Fig. 2:**
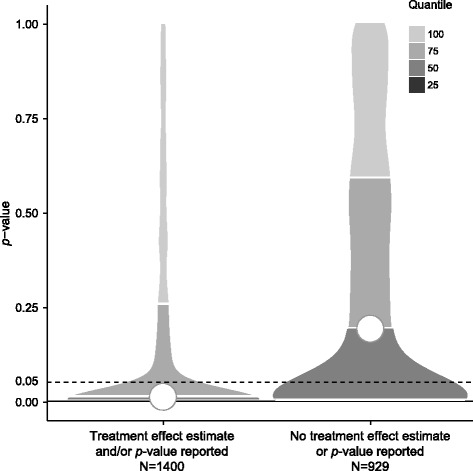
Density plot of *p*-values for trials with results for the first primary outcome posted at ClinicalTrials.gov. *Left*: 1400 trials with treatment effect estimate and/or *p*-value reported. *Right*: 929 trials with no treatment effect estimate or *p*-value reported (*p*-value calculated from data reported per arm at ClinicalTrials.gov). The lowest 25 quantiles are not visible because of clustering at *p* = 0.001 and *p* = 0.005, respectively. The *white circle* indicates the median *p*-value

When a treatment effect estimate and/or *p*-value was reported (*N* = 1400 trials), results were reported as statistically significant for 844 trials (60%), with a median *p*-value of 0.01 (Q1-Q3: 0.001–0.26) (Fig. 2). For trials with no treatment effect estimate or *p*-value reported at ClinicalTrials.gov (*N* = 1423), we could calculate treatment effect estimate and the corresponding *p*-value for 929 (65%). From data posted at ClinicalTrials.gov, *p*-values could not be calculated for 494 trials (35%), mainly because of the reporting of percentages only for binary outcomes (*n* = 187), no reporting of mean (±SD) for continuous outcomes (*n* = 95), censored data (*n* = 61), and repeated measurements over time (*n* = 52). Characteristics did not differ between trials for which we could calculate *p*-values and those with no treatment effect estimate or *p*-value reported (Appendix). Among the 929 trials for which we could calculate *p*-values, results were statistically significant for 342 (37%), with a median *p*-value of 0.19 (Q1-Q3: 0.005–0.59) (Fig. 2).

### Factors associated with reporting statistically significant results for the first primary outcome

Table 2 compares trial characteristics by whether statistically significant results were reported or not. Trials with significant results reported were more likely to be phase 3 trials (74% versus 53%, *p* < 0.0001), to have an inactive comparator (64% versus 40%, *p* < 0.0001), to have an industry sponsorship (totally or partially) (88% versus 74%, *p* < 0.0001), to be subject to the FDAAA (70% versus 61%, *p* < 0.0001), and to have a larger sample size or to be a multicenter trial involving several countries than those reporting a *p*-value ≥0.05 or not reporting treatment effect estimate or *p*-value.

**Table 2:** Characteristics of completed phase 3 or 4 randomized clinical trials with a superiority hypothesis according to whether significant results (i.e., *p*-value <0.05 or treatment effect with 95% CI not including the null value) were reported at ClinicalTrials.gov

Characteristics	Categories	Trials with significant results reported (*N* = 844)	Trials with non statistically significant results or no treatment effect estimate or *p*-value reported (*N* = 1979)	*p*-value
Phase of the study	Phase 3	622 (74)	1054 (53)	<0.0001
	Phase 4	222 (26)	925 (47)	
Intervention type	Drug	675 (80)	1427 (72)	<0.0001
	Mixed interventions	97 (11)	231 (12)	
	Biological	29 (3)	115 (6)	
	Device	22 (3)	115 (6)	
	Other	21 (2)	91 (5)	
Control group	Placebo or no treatment	543 (64)	795 (40)	<0.0001
	Active treatment	301 (36)	1184 (60)	
Sponsorship	Industry totally or partly	744 (88)	1473 (74)	<0.0001
	Academic only	100 (12)	506 (26)	
Countries	Single country	361 (43)	1267 (64)	<0.0001
	Multiple countries	353 (42)	507 (26)	
	Not reported	130 (15)	205 (10)	
Location	At least one site in the USA	493 (59)	1175 (59)	0.003
	No site in the USA	221 (26)	599 (30)	
	Not reported	130 (15)	205 (10)	
Subject to the FDAAA	Yes	588 (70)	1206 (61)	<0.0001
	No	201 (24)	732 (37)	
	Not reported	55 (6)	41 (2)	
Centers	Multicenter	581 (69)	1044 (53)	<0.0001
	Single center	133 (16)	730 (37)	
	Not reported	130 (15)	205 (10)	
Sample size	Median (Q1-Q3)	305 (139–541)	179 (62–405)	<0.0001
Time to results first received at ClinicalTrials.gov (month)	Median (Q1-Q3)	13.0 (11.6–26.3)	16.5 (12.0–30.9)	<0.0001

Data are presented as n (%) unless indicated. *FDAAA* US Food and Drug Administration Amendments Act, Q1-Q3: Quartile 1-Quartile 3

On adjusted multivariate analysis, reporting statistically significant results at ClinicalTrials.gov for the first primary outcome was associated with being a phase 3 trial (odds ratio [OR] = 1.68, 95% CI 1.38–2.04), having an inactive comparator (OR = 2.19, 95% CI 1.83–2.61), having an industry sponsorship (OR = 1.57, 95% CI 1.22–2.03), and involving multiple countries (OR = 1.58, 95% CI 1.29–1.93) (Table 3).

**Table 3:** Factors independently associated with reporting statistically significant results (i.e., reporting a *p*-value <0.05 or treatment effect with 95% CI not including the null value) for the first primary outcome posted at ClinicalTrials.gov for completed superiority clinical trials

Characteristics		Adjusted OR (95% CI)	*p*-value
Phase of the trial	Phase 3 versus 4	1.68 (1.38–2.04)	<0.0001
Intervention	Drug versus other	1.20 (0.97–1.48)	0.10
Control group	Inactive versus active	2.19 (1.83–2.61)	<0.0001
Sponsorship	Industry (totally or partially) versus academic	1.57 (1.22–2.03)	0.0005
FDAAA	Subject versus not subject	1.15 (0.94–1.41)	0.17
Countries	Multiple countries versus single country	1.58 (1.29–1.93)	<0.0001
	Not reported versus single country	1.50 (1.14–1.98)	0.004
Sample size	For an increase of 100 patients	1.002 (0.99–1.005)	0.20

*OR* odds ratio, *CI* confidence interval, *FDAAA* US Food and Drug Administration Amendments Act

## Discussion

Nearly half of the studies with results posted at ClinicalTrials.gov are not required to do so as they do not seem to be subject to the FDAAA. Nevertheless, more attention should be paid to having more complete and transparent results posted. Only half of the completed trials with results posted at ClinicalTrials.gov report a treatment effect estimate and/or *p*-value for the first primary outcome posted, with 60% of these trials reporting significant results. In contrast, the proportion of trials with significant results seemed much lower when no treatment effect estimate or *p*-value was reported. Factors independently associated with reporting statistically significant results at ClinicalTrials.gov were being a phase 3 trial, using an inactive comparator, having industry sponsorship, and involving multiple countries.

ClinicalTrials.gov is the most widely used clinical trial registry worldwide, with studies registered from 190 countries, and for now it is the only one allowing standardized posting of results [17, 18]. Clinical.Trials.gov represents a crucial source of information on trial results. Previous studies showed that it allows access to results not yet published and to more complete results than in corresponding published articles [24–26]. A recent study has compared results posted at ClinicalTrials.gov to corresponding FDA reports for new drug approval trials and found large concordance between both sources [27].

In this study, we found that results of many trials are posted although not required, because nearly half of the trials with results posted did not seem to be subject to the FDAAA according to the algorithm developed by the US National Library of Medicine. This is encouraging and highlights a willingness to give access to study results for the sake of transparency and not only because it is compulsory.

Several methodological studies have evaluated compliance with the FDAAA requiring posting of results for applicable trials [28–30] but few have looked at the nature of the results posted. Only one research note evaluated an association between changes in primary outcomes and reporting a significant result [31].

Our results show that, despite the FDAAA requirement to post results from scientifically appropriate tests of statistical significance, only half of the trials with results posted reported a *p*-value and/or a measure of treatment effect. For the other trials, we attempted to calculate treatment effect estimate and *p*-values but this was not possible for 35% of trials, mainly because of insufficient reporting or because individual patient data were required (i.e., for analysis of censored outcomes or repeated measurements over time). Therefore, for about one third of trials, systematic reviewers may be unable to use the results reported at ClinicalTrials.gov when treatment effect estimates or *p*-values are not reported. Our results also suggest that the proportion of trials with significant results differs between trials reporting or not a treatment effect estimate and/or *p*-value. Although we could not calculate treatment effect and corresponding *p*-values for all trials not reporting these, our results suggest a much lower proportion of trials with significant results when no treatment effect estimate or *p*-value is reported. These results may reflect a selective posting of treatment effect estimate and/or *p*-value when results are statistically significant. This lack of transparency may have consequences when interpreting the results posted at ClinicalTrials.gov because physicians and decision-makers may be more likely to rely on trials with *p*-values already reported.

We identified some factors associated with reporting significant results at ClinicalTrials.gov. Reporting of significant results for the first primary outcome posted were more frequent for trials sponsored by industry than academic sources, which is consistent with previous studies finding that industry trials are more likely than public trials to report significant results in published articles [32, 33] and at ClinicalTrials.gov [31]. In addition, reporting of significant results was also more common for phase 3 trials and for trials with an inactive control. This finding is not surprising for trials with inactive control treatment because treatment effect estimates are expected to be higher in this situation than when the experimental intervention is compared to an active treatment.

This study has important implications. It highlights the importance of having complete results posted, including the posting of treatment effect estimate and/or *p*-value, to avoid any misinterpretation about the benefits of interventions. Because of the poor compliance with these requirements, it becomes necessary to improve their implementation. Systematic checking of results posted and automatic mailing to responsible parties may help improve the completeness of results posted. A recent article showed that sending emails to responsible parties of completed trials that do not comply with the FDAAA legal requirement to post results significantly improved the posting rate at 6 months [34]. In April 2014, the European Union voted to adopt the Clinical Trials Regulation, which requires the registration of all clinical trials conducted in Europe and the posting of trial summary results in the European Clinical trials Database (EudraCT) within 1 year after trial completion [35, 36]. This is a crucial step toward more transparency and this initiative should take advantage of the body of literature available for ClinicalTrials.gov and compliance with the FDAAA during the implementation process.

Our study has some limitations. We only looked at the first primary outcome with results posted, so our study is not representative of all results posted at ClinicalTrials.gov. For trials with no treatment effect estimate or *p*-value reported, we attempted to calculate the treatment effect and corresponding *p*-values from data reported per arm, adopting the viewpoint of systematic reviewers. However, we could not do this for about one third of trials with no treatment effect estimate or *p*-value reported because of insufficient elements reported or because the data were repeated measures or censored, situations for which individual participant data are necessary. Although there was no difference between trials for which we could calculate a *p*-value and those with no treatment effect estimate and/or *p*-value, our results cannot be extrapolated to all trials with no treatment effect estimate or *p*-value reported. Finally, we focused on results reported at ClinicalTrials.gov and did not determine whether results had been published or not.

## Conclusions

Only half of completed trials with results posted at ClinicalTrials.gov had a treatment effect estimate and/or *p*-value reported for the first primary outcome, with significant results in 60% of these. The proportion of trials with significant results seemed much lower when no treatment effect estimate or *p*-value is reported, which may suggest a selective posting of treatment effect estimate and/or *p*-value when results are statistically significant. More efforts should be expended to improve the transparency in posting results at ClinicalTrials.gov.

## Appendix

**Table 4:** Characteristics of trials with no treatment effect estimate or *p*-value reported at ClinicalTrials.gov and those for which *p*-values could be calculated

Characteristics	Categories	Trials with no treatment effect estimate or *p*-value reported (*N* = 1423)	Trials with no treatment effect estimate or *p*-value reported for which *p*-values could be calculated (*N* = 929)
Phase of the study	Phase 3 Phase 4	728 (51) 695 (49)	440 (47) 489 (53)
Intervention type	Drug Mixed interventions Biological Device Other	1014 (71) 148 (10) 96 (7) 97 (7) 68 (5)	676 (73) 92 (10) 55 (6) 57 (6) 49 (5)
Control group	Placebo or no treatment Active treatment	516 (36) 907 (64)	347 (37) 582 (63)
Sponsorship	Industry totally or partly Academic only	1055 (74) 368 (26)	674 (73) 255 (27)
Countries	Single country Multiple countries Not reported	982 (69) 292 (21) 149 (10)	672 (72) 163 (18) 94 (10)
Location	At least one site in the USA No site in the USA Not reported	822 (58) 452 (32) 149 (10)	529 (57) 306 (33) 94 (10)
Subject to the FDAAA	Yes No Not reported	843 (59) 556 (39) 24 (2)	521 (56) 390 (42) 18 (2)
Centers	Multicenter Single center Not reported	696 (49) 578 (41) 149 (10)	437 (47) 398 (43) 94 (10)
Sample size	Median (Q1-Q3)	156 (60–378)	146 (60–372)
Time to results first received at ClinicalTrials.gov (month)	Median Q1-Q3)	17.1 (12.0–32.3)	17.8 (12.1–32.9)

Data are presented as n (%) unless indicated. *FDAAA* US Food and Drug Administration Amendments Act, Q1-Q3: Quartile 1-Quartile 3

## Abbreviations

CI: confidence interval
FDA: Food and Drug Administration
FDAAA: Food and Drug Administration Amendments Act
ICMJE: International Committee of Medical Journal Editors
OR: Odds ratio
Q1: Quartile 1
Q3: Quartile 3
RCT: Randomized controlled trial
SD: Standard deviation
SE: Standard error
